# Pragmatic health technology appraisals of biosimilars: a pilot case study of bevacizumab in colorectal cancer for NICE

**DOI:** 10.1186/s12962-026-00761-w

**Published:** 2026-05-12

**Authors:** Matthew D. Stevenson, Mon Mon-Yee, Jessica E. Forsyth, Amir Montazeri, Mark P. Saunders

**Affiliations:** 1 Sheffield Centre for Health and Related Research, University of Sheffield, Regent Court, 30 Regent Street, Sheffield, S1 4DA UK; 2 The Clatterbridge Cancer Centre NHS Foundation Trust, Wirral, CH63 4JY UK; 3 The Christie, Wilmslow Road, Manchester, M20 4BX UK

**Keywords:** Metastatic colorectal cancer, Economic evaluation, Biosimilars, Bevacizumab

## Abstract

**Background:**

The introduction of biosimilars can lead to substantial price reductions which can alter cost-effectiveness conclusions in health technology assessments. This study, undertaken for the National Institute for Health and Care Excellence (NICE), aimed to pilot a test-and-learn approach for biosimilars appraisals whilst pragmatically assessing the cost-effectiveness of bevacizumab (originator and biosimilars) plus fluoropyrimidine-based chemotherapy versus chemotherapy alone for patients with metastatic colorectal cancer.

**Methods:**

A partitioned survival model with three health states (progression-free, post-progression and dead) was used. No additional active lines of treatment were modelled assuming that all further treatments were cost-effective and assuming no interaction between bevacizumab and the efficacy of subsequent treatments. Analyses were undertaken independently for the use of bevacizumab plus fluoropyrimidine-based chemotherapy as first-line, and second-line, treatment. Analyses were conducted in line with NICE guidance. The clinical effectiveness of treatments was estimated from reviews of previous NICE technology appraisals, published literature, and expert input. Confidential prices of bevacizumab weighted by the market share were used.

**Results:**

The use of bevacizumab plus fluoropyrimidine-based chemotherapy was shown to extend life and increase quality-adjusted life-years (QALYs). Whilst cost per QALY values cannot be reported due to confidentiality reasons, the NICE Appraisal Committee stated that these were below what NICE considers a cost-effective use of resources for both first- and second-line treatment.

**Conclusions:**

The pragmatic modelling approach piloted was accepted by NICE and allowed for a quicker, internally funded, appraisal of bevacizumab biosimilars; this approach likely can be extended to other biosimilar appraisals.

**Supplementary Information:**

The online version contains supplementary material available at 10.1186/s12962-026-00761-w.

## Background

The loss of exclusivity for an intervention is typically associated with significant price decreases when generics or biosimilars enter the market. A report from Oxera indicates that the price of a generic drug is 30% of the price of the originator prior to loss of exclusivity on entry and falls to 10% between two and five years after loss of exclusivity [1]. These price decreases could mean that interventions that were not recommended for routine use on grounds of cost-effectiveness may be cost-effective under the revised pricing. Previous research has indicated that health technology assessments for biosimilars would be relevant when the originator is not reimbursed [2]. This appears logical for safe and clinically effective treatments.

In England, the National Institute for Health and Care Excellence (NICE) needs to appraise interventions in line with its manual [3] and treats biosimilars as equivalent to the originator product. A potential barrier to an appraisal is the fee requested by NICE, which in March 2026 was £186,100 for a large company with a product undergoing a single technology assessment as it is unlikely that this would be paid by manufacturers of biosimilars [4]. In the pilot multiple technology assessment appraisal of bevacizumab for metastatic colorectal cancer (mCRC), which is the focus of this paper, this cost was centrally-funded, by NICE using core-funding received from the Department of Health and Social Care. The scope and matrix for this research are available online [5].

We, in our role of External Assessment Group, were requested to (1) conduct a pilot process for biosimilar appraisals using a test-and-learn approach and (2) review the clinical effectiveness evidence and develop a health economic model, using a pragmatic approach, to assess the cost-effectiveness of bevacizumab (originator and biosimilars) with fluoropyrimidine-based chemotherapy compared with fluoropyrimidine-based chemotherapy alone. The timelines were shorter than for standard multiple technology appraisals. As expected, no company submissions were received, and NICE restricted the population to patients who were not candidates for targeted treatments or immunotherapies. This paper sets out the principles and assumptions used in our case study. Whilst the cost-effectiveness results are confidential, the methodology employed can be freely reported. The learnings from this evaluation are intended to inform NICE’s ‘Whole Lifecycle Approach’, which ensures recommendations are readily accessible, clear, practical and kept up to date with the latest evidence [6–8]. The approach aims to shift from relatively static recommendations to more dynamic resources that evolve over time, while widening access to cost-effectiveness treatments as new evidence emerges. Prior to this work, as a company would not have funded an updated assessment, the negative recommendations previously issued by NICE would have remained, precluding the use of biosimilar bevacizumab as first- and second-line treatments [9, 10]. 

## Methods

### Clinical-effectiveness and duration of treatment

Given the expedited approach we did not undertake a formal systematic review to estimate the clinical effectiveness of bevacizumab in mCRC but instead reviewed NICE’s previous technology appraisals relating to mCRC and sought advice from clinicians regarding any new studies, which are summarised later. Real world data were not considered as they would not provide comparative evidence. Bevacizumab had previously been appraised by NICE for use in first-line treatment of mCRC in TA118 in combination with FOLFIRI [9], and in TA212 (in combination with FOLFOX and CAPOX) [10]. FOLFIRI consists of 5-fluorouracil (5-FU), folinic acid (FA) and irinotecan; FOLFOX consists of 5-FU, FA and oxaliplatin; and CAPOX consists of oxaliplatin and capecitabine. An appraisal of second-line treatment of bevacizumab (in combination with FOLFIRI) in mCRC is reported in TA242 [11]. 

Additionally, two meta-analysis papers covering the use of bevacizumab with fluoropyrimidine-based chemotherapies as first- and second-line treatments were identified [12, 13]. The meta-analyses reported by Mocellin et al. [13] compared the second-line use of bevacizumab plus chemotherapy versus chemotherapy alone and synthesised results from four trials. The meta-analyses synthesised hazard ratios for progression-free survival (PFS) and overall survival (OS) and these hazard ratios were used in a sensitivity analysis. Time-to-event data were extracted or estimated from the studies deemed most appropriate to use within the decision problem. Digitised Kaplan-Meier (KM) curves from study publications were used to generate pseudo-individual patient data for OS and PFS using the algorithm reported by Guyot et al. [14]. The exception was one study (NO16966) [15] submitted to NICE as part of TA212 where KM estimates of OS and PFS were provided in confidence. Seven parametric survival models were fitted to the time-to-event data, which were the exponential, Weibull, Gompertz, log-normal, log-logistic, gamma and generalised gamma distributions. The survival model selection process included: (i) examination of Akaike Information Criterion and Bayesian Information Criterion, (ii) visual inspection of the fitted survival models against the KM survival functions and (iii) consideration of the clinical plausibility of the survival model predictions (based on hazard plots and input from clinical experts). Empirical hazard plots of the trial data were visualised using piecewise exponentials (via the *pehaz* function in R) and hazard functions smoothed using b-splines (via the *bshazard* function in R).

When choosing survival models we used a pragmatic approach, balancing between providing sufficient information for the Appraisal Committee to make an informed decision and limiting the number of analyses run. Clinical input was used to select the most-appropriate long-term hazards. Where there was little difference in the goodness-of-fit statistics between models, the model which had the largest difference compared to the base case selection (in terms of long-term hazards) was chosen for sensitivity analyses.

Time to treatment discontinuation (TTD) was estimated using the KMs for time on treatment, where available, (Study NO16966 [15] for first-line FOLFOX/CAPOX-containing regimens). In the absence of time on treatment KM data, mean treatment durations were obtained and assumed applicable (Study AVF2107g [16] for first-line FOLFIRI-containing regimens and from Study E3200 [17] for FOLFOX/CAPOX-containing second-line regimens). Based on the data from Study E3200, treatment durations in the second-line setting were estimated as 5 months for bevacizumab plus FOLFOX/CAPOX and 4 months for FOLFOX/CAPOX alone. The mean durations of each regimen component in the first-line setting are confidential and cannot be presented.

### Cost-effectiveness

Due to the pragmatic approach required, we did not consider developing a fully sequential treatment model including interventions used later in the mCRC treatment pathway. Instead, two simplifying assumptions were used such that our approach would provide indicative estimates of the incremental cost-effectiveness ratio (ICER), which was defined in terms of cost per quality-adjusted life year (QALY) gained. The first assumption was that subsequent NICE-recommended treatments after the relevant treatment line are cost-effective. Thus, extending the life of a person with first-line bevacizumab treatment would not result in patients receiving treatments that were not cost-effective and produce perverse results from a cost-effectiveness perspective. The second assumption was that there is no interaction between the efficacy or duration of subsequent treatments and the use of bevacizumab treatment at an earlier line. Thus, the possibility that the early use of bevacizumab would dilute the effectiveness of a subsequent treatment has not been explicitly considered. Whilst this may be a limitation, this would not be a problem if interventions at later lines remain cost-effective, despite some dilution of effectiveness. In our case study, clinicians commented that the previous use of bevacizumab had not statistically significantly affected the efficacy of subsequent bevacizumab based on data presented by Masi et al. [18]. and Prager et al. [19]. and thus it was deemed that this assumption held.

If both of these assumptions hold then a partition survival model for a last-line treatment is deemed to be appropriate, In this model patients reside in one of three health states: progression-free disease, progressed disease and dead, which are used to estimate the costs and QALYs associated with each treatment option (see Appendix 1, Figure S1). The numbers of patients in each health state are determined by the extrapolations of OS and PFS distributions. The proportion above the OS distribution determines the proportion of patients who have died, the proportion under the PFS distribution determines the proportion of patients who remain progression-free and alive, and the remaining proportion of patients are assumed to be alive and have progressed disease. All costs that would be incurred and QALYs gained due to subsequent treatments are intentionally excluded from the model on the premise that subsequent treatments would only increase the incremental net monetary benefit (iNMB) as these treatments are cost-effective (through assumption 1) and there is no interaction between their relative effectiveness and the early use of bevacizumab (through assumption 2).

Excluding subsequent treatments which extend life from the model results in an underestimation of life expectancy (and QALYs gained) associated with standard of care. The discounted QALYs associated with standard care is an important component in determining whether a disease severity modifier weighting should be used which can alter the cost per QALY threshold used in an appraisal [8]. Underestimating the QALYs generated by standard care could result in an inappropriately high disease severity modifier being used. We attempted to address this by adding the QALYs estimated from the results presented in a NICE appraisal of later-line mCRC treatments [20] to the QALYs generated in our models of first-line and second-line use of bevacizumab. For further information on the methods used, see Sect. 5.5 and Table 42 of the EAG report contained in the NICE committee papers [21]. The approach used, however, has limitations. Firstly, it allows for double counting of QALYs accrued during the period of time after a patient progresses in the simple partition survival model to death, as there will be QALYs associated with both the initial treatment line (where the patient is in the progressed disease state) and the subsequent treatment where the patient would be in the progression-free state. A second limitation is that all PFS events are assumed to be progression rather than death and therefore all patients are assumed to receive subsequent-line treatment. Both limitations are likely to overestimate the QALYs gained under current care and could be unfavourable to an intervention if the proportional or absolute QALY shortfall were marginally below the threshold required for an increased QALY weight. In this report, we have run results at second-line using disease severity modifier weights of both 1.0 and 1.2. Both deterministic and probabilistic base case ICERs were generated as were deterministic scenario analyses.

### Population of key model inputs

Patients receiving first-line treatment were assumed to be 60 years old, with 40.0% of the cohort female, while those receiving second-line treatment were assumed to be 61 years old with 39.5% of the cohort female, based on average values from Studies NO16966 [15] and AVF2107g [16]. 

For first-line treatment the utility for the progression-free health state was 0.77 and was 0.68 for the progressed health state in line with TA212 [10]. For second-line treatment, the utility values from TA1008 [20] were used, which were 0.73 for patients in the progression-free health state and 0.64 for patients with progressed disease. All utilities were assumed to decrease as patients increased in age following Hernández Alava et al. [22]. The higher utility for patients who have progressed after first-line treatment in the second-line model is assumed to reflect the situation where only fitter patients, with higher utilities, will receive second-line treatment. Disutilities (and costs) associated with treatment-related adverse events were included in the model based on observed data from the key studies and on targeted literature reviews, but these corresponded to relatively small values. No caregiver disutility was assumed as we did not consider these to be of exceptional magnitude compared with any treatments that may be displaced should bevacizumab be recommended.

Our analyses use the mean Medicines Procurement and Supply Chain price of the biosimilar and seven originator drugs weighted by the confidential market share across NHS trusts, with sensitivity analyses using the weighted median cost. We used a distribution of body weight/body surface area and the relative dose intensities reported in the studies to estimate the percentages of patients requiring each number of vials. Vial sharing was not assumed in the base case but explored in sensitivity analyses.

For model simplicity, resource use was assumed to be identical in both first-and second-line settings for all treatment regimens. Resource use data for drug administration, monitoring and disease management were taken from Study NO16966 [15], and Study AVF2107g [16]. Unit costs were sourced from the NHS Reference Costs 2023/24 [23] and PSSRU 2024 [24]. which were the most current at the time of the analysis. The expected monthly costs of drug administration and monitoring were estimated separately for each treatment, ranging from £819 to £1,527 (see Table 24 of TA1136 committee papers) [21]. Patients receiving bevacizumab in combination with fluoropyrimidine-based chemotherapy required additional resources including hospital-based pharmacists and nurses, resulting in slightly higher costs than for patients receiving fluoropyrimidine-based chemotherapy alone. In both the first-line and second-line model, the costs of being in the progression-free health state was estimated to be £133 per month, increasing to £273 for those in the progressed disease state. Terminal care costs were assumed to be £6,265 based on the value reported by Round et al. [25]. inflated to 2023/24 prices and applied as a once-only cost for all patients upon entry into the dead state.

## Results

### Clinical effectiveness

Following our pragmatic review, two clinical studies (NO16966 [15] and AVF2107g [16]) were selected to inform the economic analysis in the first-line setting and one study (E3200) in the second-line setting. Study NO16966, the pivotal study for TA212 [10], evaluated bevacizumab plus FOLFOX/CAPOX compared with FOLFOX/CAPOX alone, while Study AVF2107g, the pivotal study for TA118 [9], evaluated bevacizumab plus IFL versus IFL and placebo. IFL comprises 5-FU, FA/leucovorin and irinotecan and is henceforth referred to as “FOLFIRI” in keeping with the nomenclature used in TA118. Study E3200 [17] assessed bevacizumab plus FOLFOX compared with FOLFOX alone. In Study NO16966, analyses were based on the pooled data from the FOLFOX- and CAPOX-containing arms of the 2 × 2 factorial design, assuming equivalent efficacy between regimens; this assumption was supported by clinical expert opinion. In Study AVF2107g, OS estimates may be subject to confounding due to continued use of bevacizumab beyond progression in the bevacizumab plus IFL arm. Additionally, Study AVF2107g used a bolus 5-FU and FA regimen (5-FU and FA/leucovorin administered as a weekly intravenous [IV] bolus injection for four weeks in a six-week treatment cycle), whereas the 5FU and FA in the FOLFIRI regimen, is infusional (5-FU and FA initially given as a short infusion followed by a 46-hour IV infusion every two weeks) and is more commonly used in the NHS. We are unclear of the likely impact of these limitations on cost-effectiveness. Study E3200 excluded patients previously treated with oxaliplatin or bevacizumab, however, this is unlikely to have a significant impact on the economic model because prior treatments were not explicitly considered in the economic analysis for the second-line setting. No studies were identified assessing the use of bevacizumab plus FOLFIRI in second-line setting. As summarised in Table 1, all studies indicate that the addition of bevacizumab to the fluoropyrimidine-based chemotherapy resulted in a statistically significant increase in both PFS and OS.

**Table 1:** Summary of the differences in progression-free and overall survival from clinical studies

	Difference in median PFS / OS (months)	Hazard ratio, *p* value	Source
***PFS_ first-line setting***			
Bevacizumab plus FOLFOX/CAPOX vs. FOLFOX/CAPOX alone	1.4	HR = 0.83, *p* = 0.0023	NO16966(15)
Bevacizumab plus FOLFIRI vs. FOLFIRI alone	4.4	HR = 0.54, *p* < 0.001	AVF2107g(16)
***OS_ first-line setting***			
Bevacizumab plus FOLFOX/CAPOX vs. FOLFOX/CAPOX alone	1.4	HR = 0.89, *p* = 0.0769	NO16966
Bevacizumab plus FOLFIRI vs. FOLFIRI alone	4.7	HR = 0.66, *p* < 0.001	AVF2107g
***PFS_ second-line setting***			
Bevacizumab plus FOLFOX/CAPOX vs. FOLFOX/CAPOX alone	2.6	HR = 0.61, *p* < 0.0001	E3200(17)
***OS_ second-line setting***			
Bevacizumab plus FOLFOX/CAPOX vs. FOLFOX/CAPOX alone	2.1	HR = 0.75, *p* = 0.0011	E3200

FOLFOX: folinic acid plus fluorouracil plus oxaliplatin; CAPOX: capecitabine plus oxaliplatin; FOLFIRI: folinic acid plus fluorouracil plus irinotecan; HR: hazard ratio; OS: overall survival; PFS: progression-free survival

Using the precedent set in TA212 and clinical expert input, the efficacy of FOLFOX and CAPOX (with or without bevacizumab) was assumed to be equivalent. Therefore, survival estimates for FOLFOX/CAPOX were informed by Study NO16966 in the first-line setting and Study E3200 in the second-line setting.

In some cases, the hazard plots generated using the reconstructed pseudo-individual patient data, appeared to have a turning point. This was evident in the PFS hazard functions in the bevacizumab plus FOLFOX/CAPOX, FOLFOX/CAPOX alone and FOLFIRI alone groups in first-line treatment (see Figs. 13 and 14 of TA1136 committee papers) [21]. However, clinical experts expressed their belief that the hazard trend would likely continue to increase for both PFS and OS in both first- and second-line treatment. Therefore, we prioritised the use of models with monotonically increasing hazard trends in the base case and explored the use of alternative models in scenario analyses. Based on NICE Decision Support Unit Technical Support Document 14 [26], we additionally prioritised selecting the same type of models across treatment arms. The base case and sensitivity analyses are deemed to provide a plausible range in the ICER.

The distributions used within the base case for OS and PFS are as follows: gamma for FOLFOX/CAPOX-containing regimens (for both first- and second-line treatments) and Weibull for FOLFIRI-containing regimens. In sensitivity analyses these were changed to: log-logistic for FOLFOX/CAPOX-containing regimens (for both first- and second-line treatments) and generalised gamma for FOLFIRI-containing regimens. Fully specified models for all comparisons cannot be provided for confidentiality reasons although, for illustration, the model-predicted PFS and OS for bevacizumab plus FOLFIRI and FOLFIRI alone when used in first-line for the base case and the sensitivity analysis are shown in Appendix 1 (Figures S2 and S3). The Kaplan-Meier data for this example were digitised from the AVF2107g study [16]. 

### Cost-effectiveness

The ICERs cannot be presented for all analyses due to the confidential nature of the eight tender prices for bevacizumab, however, the incremental discounted QALYs and life years gained (LYG) associated with the use of bevacizumab can be reported. Deterministic values are provided as the model appeared linear with probabilistic results being similar to the deterministic ones. The pair-wise health-related results in the base case and in scenario analyses for both first- and second-line settings are presented in Table 2.

**Table 2:** Base case and scenario analyses results in first-and second-line settings

	Incremental LYG	Incremental QALYs (discounted)
***Base case_ first-line setting***		
Bevacizumab plus FOLFOX/CAPOX vs. FOLFOX/CAPOX alone	0.260	0.169
Bevacizumab plus FOLFIRI vs. FOLFIRI alone	0.488	0.324
***Scenario analyses exploring alternative OS and PFS models_ first-line setting***		
Bevacizumab plus FOLFOX/CAPOX vs. FOLFOX/CAPOX alone^*^	0.429	0.246
Bevacizumab plus FOLFIRI vs. FOLFIRI alone^‡^	0.342	0.236
***Base case_ second-line setting***		
Bevacizumab plus FOLFOX/CAPOX vs. FOLFOX/CAPOX alone	0.241	0.159
***Scenario analysis exploring alternative OS and PFS models_ second-line setting***		
Bevacizumab plus FOLFOX/CAPOX vs. FOLFOX/CAPOX alone^*^	0.340	0.213
***Scenario analysis using HRs for PFS and OS based on Mocellin et al.(13)***		
Bevacizumab plus FOLFOX/CAPOX vs. FOLFOX/CAPOX alone	0.203	0.135

LYG: life-years gained; QALYs: quality-adjusted life-years; FOLFOX: folinic acid plus fluorouracil plus oxaliplatin; CAPOX: capecitabine plus oxaliplatin; FOLFIRI: folinic acid plus fluorouracil plus irinotecan; HR: hazard ratio; OS: overall survival; PFS: progression-free survival

*exploring log-logistic distribution

‡exploring generalised gamma distribution

In the first-line setting, when bevacizumab was added to FOLFOX/CAPOX an estimated 0.260 LYG and 0.169 discounted QALYs were generated; these values were 0.488 LYG and 0.324 discounted QALYs when bevacizumab was added to FOLFIRI. The sensitivity analyses that changed the LYG most were assuming the alternative model fits for PFS and OS which increased LYG to 0.429 and discounted QALYs to 0.246 when bevacizumab was added to FOLFOX/CAPOX but reduced LYG to 0.342 and discounted QALYs to 0.236 when bevacizumab was added to FOLFIRI.

In the second-line setting, when bevacizumab was added to FOLFOX/CAPOX an estimated 0.241 LYG and 0.159 discounted QALYs were generated. Two sensitivity analyses changed the LYG considerably. The first was assuming alternative model fits for PFS and OS which increased LYG to 0.340 and discounted QALYs gained to 0.213; the second was using the hazard ratios for PFS and OS from the meta-analyses reported by Mocellin et al. to generate the bevacizumab plus FOLFOX PFS and OS curves from the FOLFOX alone curves [13], which reduced LYG gained to 0.203 and discounted QALYs gained to 0.135. As no studies were identified comparing bevacizumab plus FOLFIRI, the cost-effectiveness for this comparison was estimated assuming that the relative difference in ICERs between bevacizumab plus FOLFOX/CAPOX versus FOLFOX/CAPOX alone and bevacizumab plus FOLFIRI versus FOLFIRI alone in the first-line setting was generalisable to the second-line setting.

The NICE Appraisal Committee concluded that, given uncertainties around the price of bevacizumab and modelling assumptions, an acceptable ICER threshold would be towards the lower end of the range between £20,000 and £30,000 per QALY gained. Note that the Appraisal Committee meeting was held on the 5th of November 2025, before the change in April 2026 of the NICE thresholds to between £25,000 and £35,000 per QALY gained.

The Appraisal Committee further concluded that the ICERs were below what NICE considers an acceptable use of NHS resources in both first-and second-line settings, with a severity modifier of 1.2 applied in the second-line setting. Despite limitations in the estimation of QALY shortfall, the Appraisal Committee considered an application of a 1.2 severity modifier in the second-line setting to be appropriate, as the approach was likely to overestimate QALYs in the standard of care arm [27]. 

## Discussion

This paper sets out a pragmatic way to estimate the cost-effectiveness of biosimilars. Simplifying assumptions were used to reduce the time and cost required for the evaluation, cognisant that the costs were not paid by a pharmaceutical company as the drug had lost its patent, however, there is a possibility these simplifications could result in inaccurate results. For example, if there were an interaction between bevacizumab and later lines of treatment which significantly reduced the efficacy of these treatments then cost-effectiveness would be over-estimated. Fortunately, this was not believed to be relevant in our case study but could cause problems in other evaluations and necessitate more complex modelling. Additionally, if NICE had, through whatever reason, recommended treatments in later lines that ultimately were not cost-effective, or interventions in common use in later lines that are not cost-effective had not been appraised by NICE, then prolonging survival in earlier lines could reduce net monetary benefit. This was not assessed in the pilot where we assumed all subsequent treatments were cost-effective. Further simplifications were needed relating to the proportion of patients that progress rather than die and in obtaining the correct disease severity modifier. In our pilot study, the appropriate comparators were those within the pivotal trials, in other circumstances different treatments may have entered the market and be used at the relevant lines within the decision problem. In these instances, indirect treatment comparisons would be needed to obtain an estimate of relative effectiveness.

A key uncertainty relates to the most appropriate price to use for the biosimilars (and originator) as in our example there were eight confidential tender prices that had a considerable range. NICE’s published methods manual [3] states that the mean cost should be used in the base case with the median cost as a sensitivity analysis. Whilst this simplification may not overly affect the ICER when biosimilar drugs are used in subsequent lines of treatment in an appraisal, it is unlikely to be appropriate when the biosimilar is the intervention being appraised. This is because these methods assume that the price of the biosimilar or originator does not correlate with volume of sales but in a competitive market the National Health Service in England is likely to buy markedly more of the cheapest biosimilars. The methods outlined in NICE’s manual is thus likely to inflate the price of the biosimilars/originator and result in higher cost per QALY estimates and potentially an incorrect negative recommendation. Following interactions with NICE, the guidance from the manual was not followed, but a confidential weighted average price based on the market share in England was used which we believe is more appropriate.

The appraisal of biosimilars is believed to be driven by the belief from stakeholders that the price reductions associated with bevacizumab biosimilars would make a clinically effective treatment cost-effective at earlier lines where it previously was not. On this basis, NICE required an independent assessment of clinical- and cost-effectiveness, which resulted in our pilot work. However, should the appraisal have resulted in negative guidance then no health gains would be generated, although the pressure to use cheaper drugs earlier may be reduced. In this case study, the pragmatic approach to modelling cost-effectiveness appeared to work well and was accepted by the NICE Appraisal Committee; this could therefore be a useful basis for future evaluation of biosimilars or generics.

## Supplementary Information

Below is the link to the electronic supplementary material.

Supplementary Material 1

## Data Availability

The data used to inform the economic model are taken from published literature or were marked confidential by the company submitting to NICE. The economic model (redacted for confidential information) is available upon reasonable request.
