# Supplementary material for: Pragmatic health technology appraisals of biosimilars: a pilot case study of bevacizumab in colorectal cancer for NICE
Source: Cost Eff Resour Alloc. 2026 May 12;24:69. doi: 10.1186/s12962-026-00761-w (PMC13220503; doi:10.1186/s12962-026-00761-w)
Supplement: Supplementary file 1 — Supplementary Material 1 [file 12962_2026_761_MOESM1_ESM.docx]

**Appendix 1**

**Figure S1: Model structure**


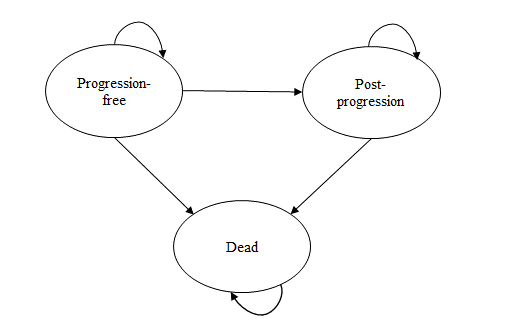


**Figure S2: A comparison of observed and model-predicted PFS for bevacizumab plus FOLFIRI and FOLFIRI alone in the first-line setting**


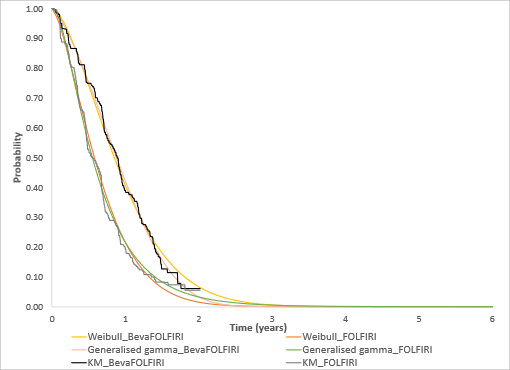


Beva: bevacizumab; FOLFIRI: folinic acid plus fluorouracil plus irinotecan; KM: Kaplan-Meier; PFS: progression-free survival; Base case – Weibull model (both arms), Scenario analysis – generalised gamma (both arms)

**Figure S3: A comparison of observed and model-predicted OS for bevacizumab plus FOLFIRI and FOLFIRI alone in the first-line setting**


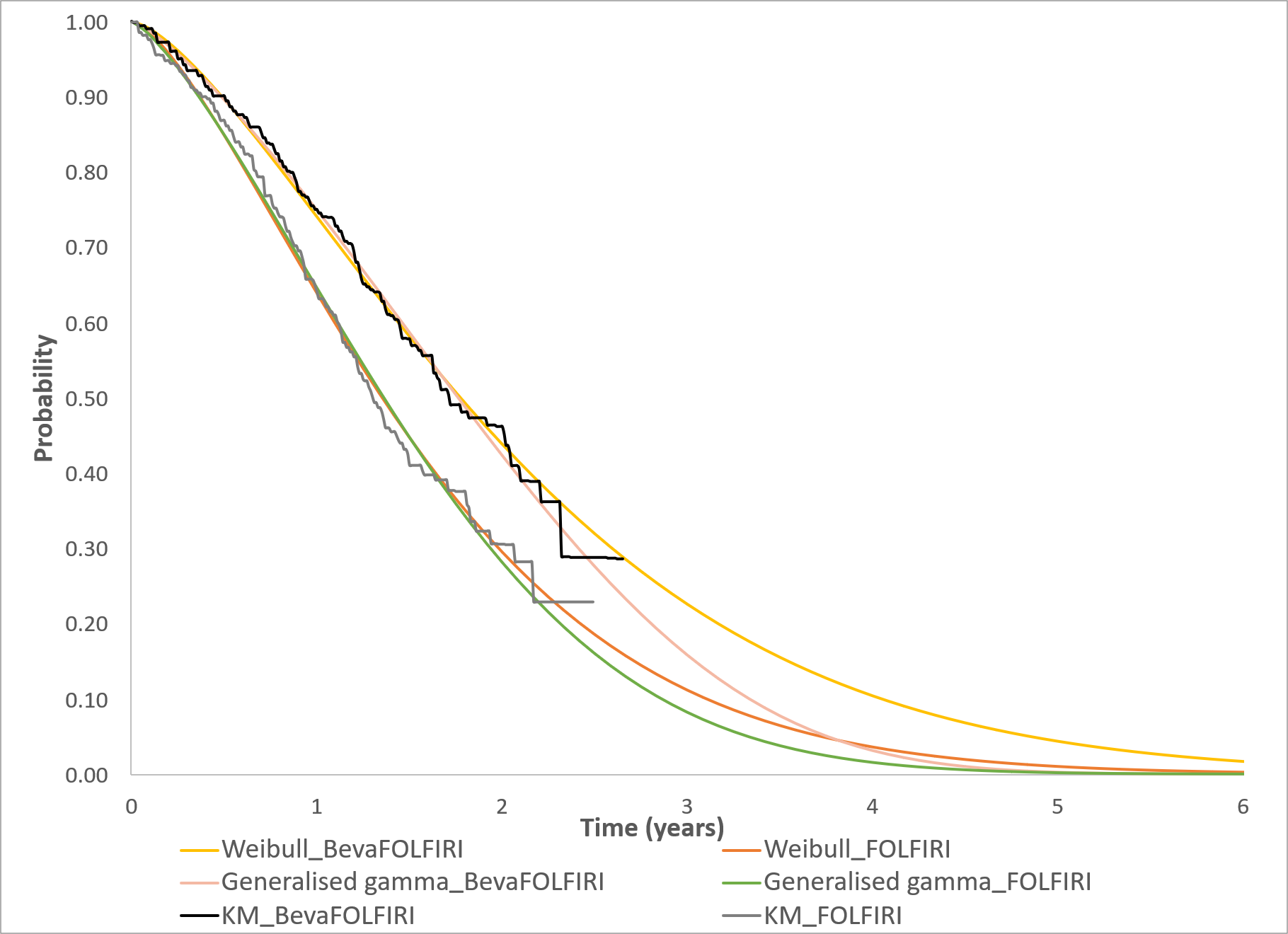


Beva: bevacizumab; FOLFIRI: folinic acid plus fluorouracil plus irinotecan; KM: Kaplan-Meier; OS: overall survival;
Base case – Weibull model (both arms), Scenario analysis – generalised gamma (both arms)
